# Design, Implementation, and Measurement Procedure of Underwater and Water Surface Antenna for LoRa Communication

**DOI:** 10.3390/s21041337

**Published:** 2021-02-13

**Authors:** Aliyu Dala, Tughrul Arslan

**Affiliations:** Integrated Micro & Nano Systems, University of Edinburgh, Edinburgh EH8 9AB, UK; t.arslan@ed.ac.uk

**Keywords:** LoRa, buoy, antenna, buffer, RSSI, gateway, IoT, water, sensor

## Abstract

There is an increasing interest in water bodies, which make up more that seventy percent of our planet. It is thus imperative that the water environment should be remotely monitored. Radio frequency (RF) signals have higher bandwidth and lower latency compared to acoustic signals. However, water has high permittivity and conductivity which presents a challenge for the implementation of RF technology. In this work, we undertook a novel design, fabrication, measurement and implementation of an antenna for a sensor node with dual ability of underwater and water surface long range (LoRa) communication at 868 MHz. It was observed that the antenna’s performance deteriorated underwater without −10 dB effective bandwidth between 668 MHz and 1068 MHz. The introduction of an oil-impregnated paper buffer around the antenna resulted in an effective 400 MHz bandwidth within the same frequency span. The sensor node with the buffered antenna was able to achieve a distance of 6 m underwater and 160 m over water surface communication to a data gateway node. The sensor node without the buffered antenna was only able to achieve 80 m over water surface communication. These experimental results show the feasibility of using the LoRa 868 MHz frequency in underwater and water surface communication.

## 1. Introduction

Water makes up a substantial part of our planet. Abundant resources could be found underwater. It is a home to more than two hundred thousand identified species of marine life [1] with around two thirds of other species yet to be identified. However, some of these species go extinct even before they are discovered [2]. This makes it pertinent to have a means to ubiquitously maintain this diversity.

The underwater environment is also of great interest to the oil and gas industries. There are many oil and gas offshore installations dotting the great oceans and seas around the world often generating prosperity and sometimes, if not properly maintained and managed through predictive and corrective maintenance, leading to devastative economic, environmental, public health, social, and community impacts [3,4,5].

Because of the very large area covered by water, this makes it a site of numerous surveys and search and recovery missions. A classic example of this is the search for the Titanic. More recently, search and recovery has involved the ill-fated Air France Flight 447 and Malaysia Airlines Flight MH370 [6]. The underwater environment, just like the terrestrial environment is home to numeral minerals such as marine nodules, polymetallic sulphides, and ferromanganese crusts [7].

Most of the activities outlined earlier may benefit from the use of a telemetry system which sends data about the status of their parameters such as location, temperature, pressure, etc. The use of wireless sensor networks helps in the relay and transmission of the obtained data from the source to destination. Different methods, both wired and wireless approaches, for the propagation of these signals underwater exist. These include acoustics [8], radio frequency (RF) [9], optical waves [10], and wired communication [11].

The underwater communication medium, unlike the terrestrial free space, has high permittivity and conductivity. These characteristics make acoustic signals better suited for long range underwater communication. However, the inherent high latency and low bandwidth of acoustics makes it unattractive for high bandwidth and real-time applications. Because acoustics operate in the hearing range of some marine life, it is injurious to them [12]. The RF signals are less successful for underwater communication because of their high attenuation and consequently limited range of transmission. However, by using several RF sensor nodes to transmit data over short distances in a multi-hop fashion, high bandwidth and low latency communication link could be achieved.

For terrestrial applications, the RF transmissions are more advantageous to acoustic signals because of its lower latency and higher bandwidth [13,14]. Thus, from the water surface to any terrestrial base station, the RF is better suited for the transmission of data.

The candidates for this RF communication can be broadly classified as short range or long range. Technologies such as Bluetooth, WiFi, and Zigbee can be grouped into the former. These would not be useful in Internet of Things (IoT) applications that require sending low bandwidth sensor data over long distances. For these, the low power wide area (LPWA) communication technologies are more suited. The two contending candidates for these technologies are the narrowband IoT (NB-IoT) and the long range (LoRa). The NB-IoT was set up by *the* 3rd Generation Partnership Project (3GPP). It is part of the long-term evolution (LTE) standard but has been stripped of some of its features such as handover, carrier aggregation, and dual connectivity to simplify it. It uses the quadrature phase shift keying (QPSK) modulation scheme and operates in the licensed band of LTE [15].

LoRa operates in the unlicensed bands below 1 GHz. It employs the chirp spread spectrum modulation (CSS) which enables it to have the long great link budget. NB-IoT boasts better quality of service (QoS), reliability, and range. However, LoRa is unlicensed, thus cheaper, and has better performances with respect to battery management and capacity [15], which are essential capabilities to a wireless sensor. We have adopted the LoRa technology for this project because of these advantages it offers.

### Literature Review

In this part, we shall review the state of the art in this field. We shall explore the different long range sensor node communication systems that were developed using the LPWA technology and review the designs that are capable of both water surface and underwater communication taking into account the effects of the water surface and underwater environment on the antenna performance.

Off-the-shelf components were used to assemble a low-cost unmanned surface vehicle to collect parametric data of rivers, lakes, or seas in [16]. The data collected were transmitted using LoRa to a cloud infrastructure. Arduino UNO was used for data acquisition and processing. A Libelium SX1272 LoRa module was used for data transmission.

In [17], the reliability of LoRa in an estuary flooding scenario was investigated using two experimental setups. One was built so that all communication was done on land, to serve as a basis for comparison, while the other was deployed in a river estuary to study the effects of the water surface and the changing water levels on the propagated signal. The antenna heights and distances of the LoRa node with the gateway were investigated. The study concluded that reliability of the LoRa communication depended on the height of the LoRa nodes and consequently the tidal levels.

The old first-generation sailing monitoring systems based on 3G technology were replaced by LoRa low power wide area network (LPWAN) in [18]. This is due to the former’s limitations with respect to coverage and high-power consumption. The experiment verified that an increase in the spreading factor (SF) or a decrease in bandwidth (BW) increases the over-the-air transmission time.

The influence of water surface on the radiation pattern of a spiral antenna operating in the S-, C-, X- and Ku-bands was investigated by [19]. A model of the spiral antenna was developed in Computer Simulation Technology (CST) Suite and the sea water was modelled by placing a perfect electric conductor (PEC) material under the antenna perpendicular to its aperture. The simulation was performed from 2 to 18 GHz. Different scenarios were adopted for the simulation. The first did not involve a water surface. Subsequently, the antenna was raised to a height of 1, 3.5, and 15 m. They concluded, in the study, that it is advantageous to raise the antenna as high as possible from the water surface to maintain the fidelity of the received signal.

A log-periodic antenna for long range communication was simulated in [20] for sea water quality monitoring. The antenna was simulated at 36 cm above the sea water surface. The sea water surface was found in this study to improve the matching of the antenna.

In [21], a bowtie antenna was implemented for underwater communication using 433 MHz frequency range. The antenna was designed based on a previous work by A.A. Abdou et al. [22]. Initially, the antenna was designed to resonate at 433 MHz in air with a return loss of around −10 dB. A 1.6 mm printed circuit board with a copper thickness of 35.6 mm and FR-4 glass-reinforced epoxy laminate was used for the design of the antenna. A 0.30 cubic meter volume was used to simulate the water surface. The work in [21] performed lab tests for the submerged sensor nodes and subsequently deployed the sensor nodes in a canal where the maximum distance achieved was 7 m and 5 m for baud rates of 1.2 kbps and 25 kbps respectively.

In our work, we designed, measured, and implemented both a sensor node that transmits water temperature data and an IoT gateway that receives this data and publishes it to the Internet. The antenna is an integral part of any wireless sensor network, thus we present a novel approach to antenna design and implementation of a braid antenna using an oil impregnated paper buffer to improve the performance of the antenna performance underwater and at water surface operating at 868 MHz frequency range. This is the authorised Industrial Scientific and Medical (ISM) band in Europe. The sensor nodes developed would be capable of operating submerged in water and on water surface.

We used the CST and measurements to show the degradation of the reflection coefficient of the antenna when placed in a polylactic acid (PLA) enclosure placed on a buoy and deployed on the water surface and when the antenna is submerged in water.

## 2. Materials and Methods

### 2.1. Antenna Design

#### Base Antenna

The initial design is a printed triangular monopole antenna (PTMA) [23]. It has a triangular patch with a coplanar waveguide (CPW) and ground on the top plane of the substrate. The dimensions of the antenna were determined by equating the lower frequency, $f_{L}$ of the PTMA and its dimensions as can be seen in Equation (1) [22]:

(1) $$f_{L}=\frac{c}{\lambda }=\frac{7.2}{\left\{\left(L+r+p\right)\times k\right\}}GHz,$$

where L and r are the height of the planar monopole antenna and the effective radius of the equivalent monopole antenna, respectively. p is the length of the 50 Ω feed line of the antenna. All these dimensions are in cm. k can be determined form $\surd \varepsilon_{ff}$. $\varepsilon_{ff}$ is the relative permittivity of the substrate used for the patch antenna design. FR-4 was used for this research.

To determine both L and r, the side length T of the antenna is used in Equations (2) and (3) [22]:

(2) $$L=\frac{\sqrt{3}}{2}T$$

$$r=\;\frac{T}{4\pi }$$

**Figure 1:**
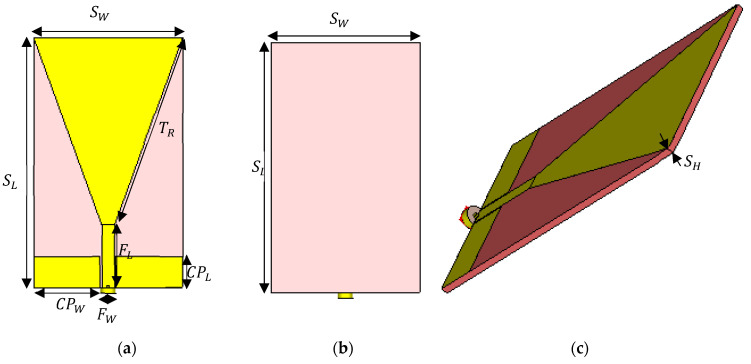
Base antenna (**a**) front (**b**) back (**c**) perspective.

Using Equations (1)–(3), the dimensions obtained for the base antenna can be seen in Figure 1 and Table 1.

A parameter sweep was performed on the substrate thickness and the dimension with the optimal value for the reflection coefficient, 2.4 mm, was chosen for the final design.

### 2.2. Braid Antenna

#### 2.2.1. Braid Antenna Simulation

**Figure 2:**
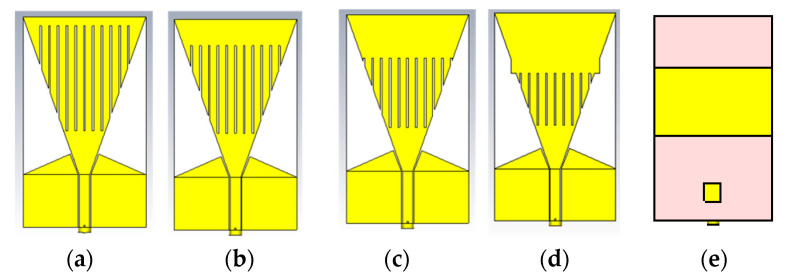
Braided antenna design; (**a**) 5 mm (**b**) 15 mm (**c**) 25 mm (**d**) 35 mm (**e**) back.

The buoyed antenna and the LoRaWAN Gateway operate at 868 MHz in Europe. Thus, the designed antenna must be able to resonate or operate at this frequency. In order to achieve that frequency of operation on the base PTMA design, some modifications were made to it. A scalene triangle was added to the CPW and 11 braid slots of 1 mm were introduced on the triangular patch antenna. Two rectangular elements were introduced on the bottom plane of the substrate as can be seen from Figure 2e. A rectangular element was introduced from the top end of the braided patch and its length was varied from 5 mm to 35 mm at a step of 10 mm. A representation of these could be seen from Figure 2a–d.

#### 2.2.2. Fabrication

**Figure 3:**
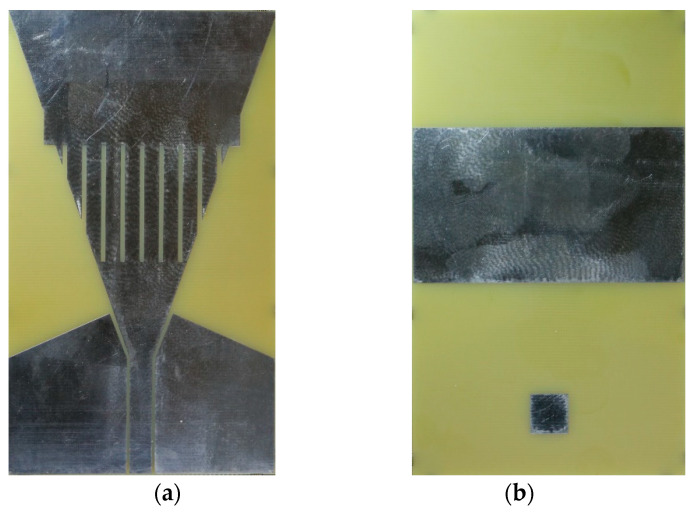
Picture of fabricated braided antenna (**a**) front (**b**) back.

The braid antenna with 35 mm connector was chosen due to its performance. The simulation results for this are shown in Section 3. The optimal substrate thickness was determined as 2.4 mm. This was obtained by performing parametric sweep of the substrate thickness in CST and the 2.4 mm thickness exhibited the best −10 dB bandwidth performance. The fabricated antenna can be seen in Figure 3. It was produced using FR-4 substrate and a copper with a thickness of 35 mico metres by Eurocircuits.

To determine the performance of the fabricated braid antenna, three LoRa antenna were used. An Eightwood Antenna, ANT-868-OC-LG antenna from Linx Technologies, and The Things Network (TTN) LoRa gateway antenna were used for benchmarking the fabricated Braid antenna.

**Figure 4:**
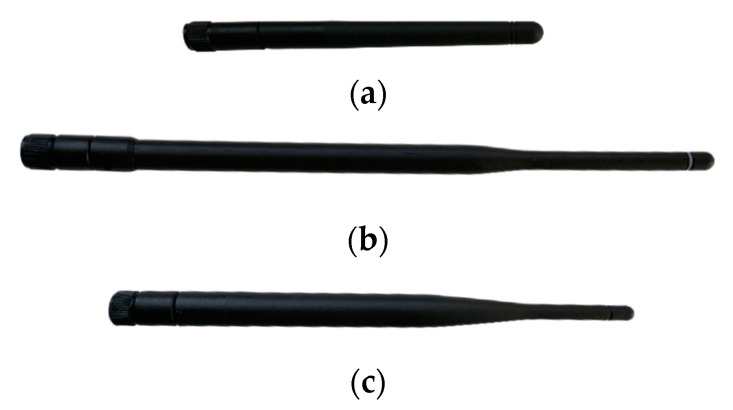
Off-the-shelf benchmark antennas (**a**) Eightwood Antenna (**b**) Mouser (**c**) Things Network Gateway.

The antennas’ reflection coefficients were measured using a PC that was connected to a HP 8753 vector network analyser (VNA) via a General Purpose Interface Bus (GPIB). The S11 was captured using a software on the PC. The VNA has a dynamic range of 100 dB with frequency range of 300 kHz to 3 GHz. Calibration sets of 3.5 mm were used to prepare the 50 ohm ports of the VNA for the measurements. The frequency was swept from 668 MHz to 1068 MHz with the centre frequency at 868 MHz, corresponding to the Europe’s ISM band. The source power was selected at −10 dBm. A TTN antenna and a pair each of the Eightwood antenna and the OC-LG antennas that were off-the-shelf, were used in the measurement. A picture of the antennas used can be seen in the Figure 4a–c.

#### 2.2.3. Received Signal Strength Indicator (RSSI) Tests

**Figure 5:**
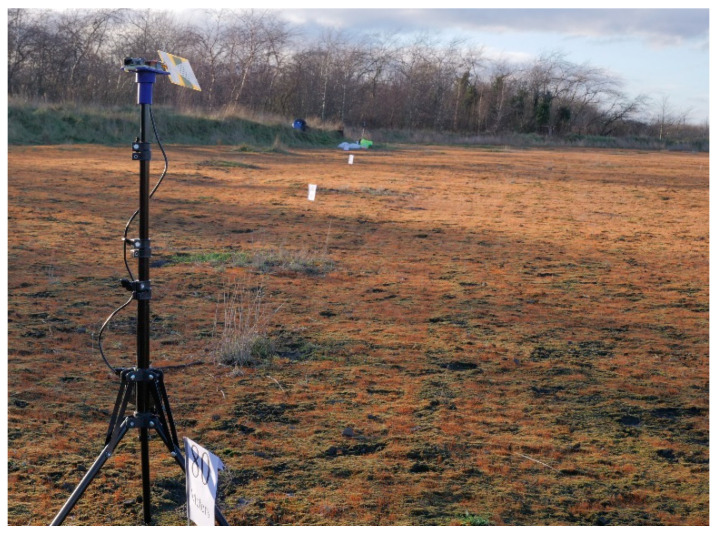
Setup for the received signal strength indicator (RSSI) tests for receive and transmit antennas.

Based on the reflection coefficient performance, the braid antenna and Eightwood antenna were compared using received signal strength indicator tests. The RFM95 LoRa module was used as the transceiver for transmitting and receiving the signal. An Atmega328 was used as the microcontroller responsible for processing the captured signal. The antennas were mounted on a tripod and a support platform that was 3D-printed. The transmitting LoRa node was equipped with a temperature sensor. The receiving antenna had an organic light-emitting diode (OLED) display that printed out the received temperature as well as the RSSI of the received signal. Both the transmitter and receiver were ensured to have a clear line of sight (LoS) to minimise both destructive and constructive interference that may result from obstacles. The transmitter was fixed at a spot while the receiver was moved at a space of 20 m. The RSSI at each point was recorded. The setup for the receiver can be seen in Figure 5. The receiver is at 80 m while the transmitter is at 0 m.

### 2.3. Systems Integration

**Figure 6:**
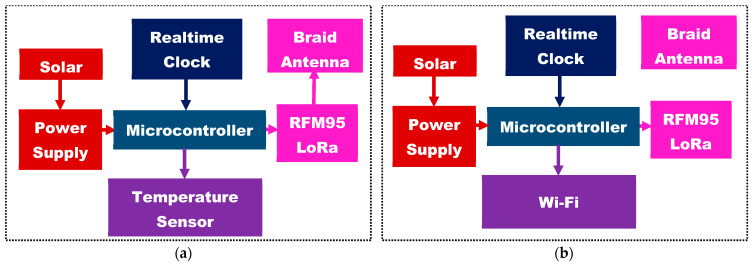
(**a**) Buoy sensor node (**b**) Internet of things (IoT) gateway.

The system comprises of a sensor node (SN) that sends water surface temperature and a terrestrial IoT gateway, that receives this signal and publishes it on the Internet. The SN was placed on a buoy and deployed on water to measure the water surface temperature and send the data to the terrestrial IoT gateway. The system architecture for the SN and IoT gateway can be seen in Figure 6a,b, respectively. The systems are both solar powered by a 6 V 2 W solar panels. An Atmega328P microcontroller controls the operation of the temperature sensor as well as the RFM95 LoRa module. The real-time clock, Ds3231, helps make the microcontroller to go to sleep and wake up to preserve energy which is of paramount importance in wireless sensor node operations.

The printed circuit boards (PCB) for the buoy sensor node and IoT gateway were designed using Eagle Autodesk software and fabricated.

**Figure 7:**
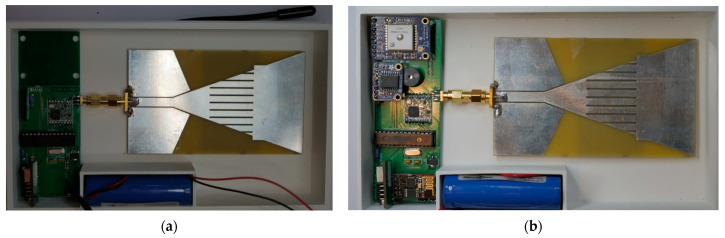
(**a**) Buoy sensor node (**b**) IoT gateway.

The enclosures for the SN and IoT Gateway were first modelled in a 3D CAD software and were fabricated using a 3D printer. A 1.75 mm PLA [24] filament was used with a nozzle diameter of 0.4 mm to build the enclosures. Figure 7a,b shows the base of the enclosures, the lithium ion batteries, the PCBs, and the braid antenna used for both the SN and IoT gateway respectively.

### 2.4. Submerged and Water Surface Antennas

In this subsection, the novel antenna design for the water surface and underwater environment is presented. It begins with the barrierless design and proceeds to the buffer design.

**Figure 8:**
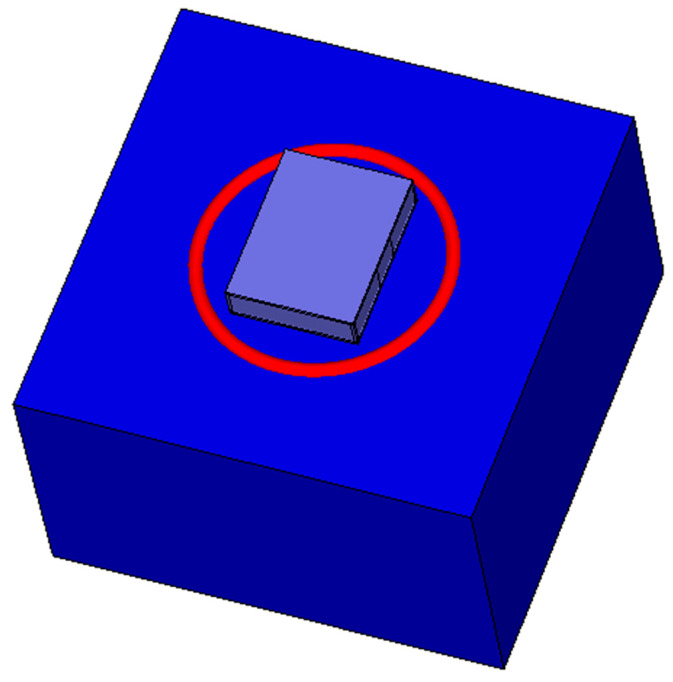
Braid antenna enclosed in PLA container on a plastic foam buoy floating on water surface.

The SN enclosure was constructed in CST and the antenna was embedded inside it. The enclosure was then placed on a torus with the characteristics of a polyethylene plastic foam, representing the buoy. A water environment was modelled, and the SN as well as the enclosure was placed on the water model. A picture of this could be seen in Figure 8. Simulation of these models resulted in a degradation of the reflection coefficient performance of the braid antenna.

#### 2.4.1. Buffer Design

In order to ameliorate the degradation of the S11 performance, a hollow barrier was constructed around the antenna using PLA with a thickness of 1 mm. The width BW and length BL of the barrier were 74 mm and 122 mm respectively. The barrier was then filled with oil-impregnated paper, so that the antenna was placed in the middle of these treated papers. A parametric sweep with respect to the barrier thickness BT was performed to determine the optimal thickness for the best S11. Four BT were used in the parametric sweep; 10 mm, 20 mm, 35 mm, and 45 mm.

#### 2.4.2. Oil-impregnated Paper Buffer

**Figure 9:**
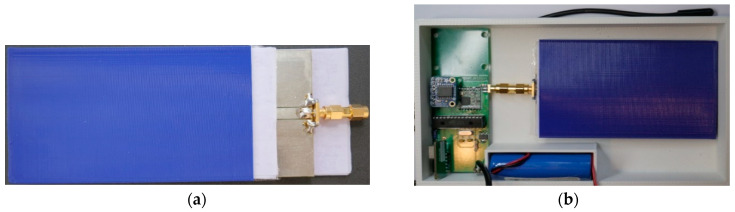
(**a**) Oil Treated paper, barrier, and braid antenna (**b**) antenna barrier inside enclosure with printed circuit boards (PCB) of sensor node (SN).

The barrier was 3D printed using PLA. A total of 30 papers were cut into a width of 36 mm and length of 120 mm so that they could fit into the fabricated barrier and the oil treated papers were used to sandwich the antenna placed in the base of the SN enclosure. These can be seen in Figure 9a,b.

### 2.5. VNA Tests

**Figure 10:**
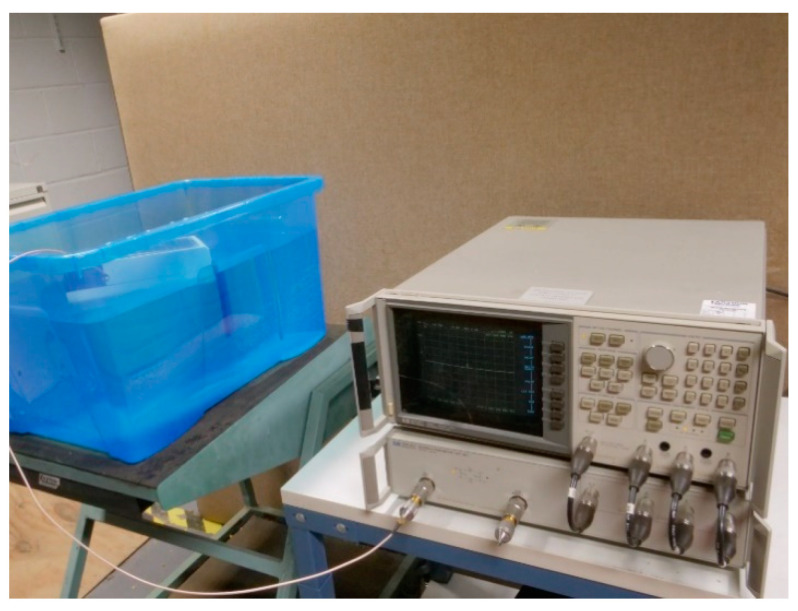
Experimental setup for antenna measurements.

The antenna with the oil-impregnated paper buffer and the one without the barrier were examined. The experimental set up for this can be seen in Figure 10. It consists of a VNA with an SMA to SMA coaxial cable connecting the antenna to the VNA through port 1 of the VNA. The results were captured using a software on the PC.

### 2.6. SN RSSI Tests

**Figure 11:**
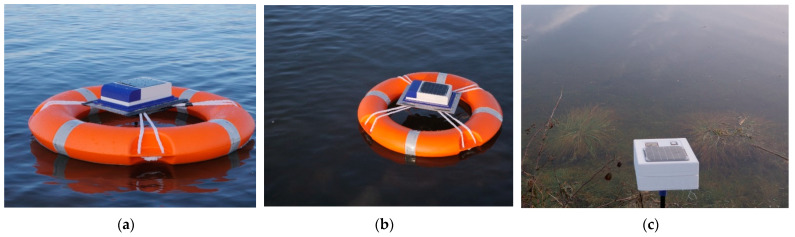
Buoyed sensor node deployed in the North Sea (**a**) with buffer, (**b**) without buffer. (**c**) Data relay node for RSSI tests.

The system level integration of the buoyed sensor node can be seen in Figure 11. The solar powered SN can be seen on the buoyed sensor node which is deployed in the North Sea. The two SN nodes were deployed.

The first one has the oil treated paper barrier (Figure 11a) while the other one does not have a buffer around the antenna (Figure 11b). This is to enable the RSSI test to determine and compare the performances of the two-system setup.

The tests for the RSSI of both the SN with the buffered antenna and the one without the barrier were undertaken in Edinburgh. The temperature sensors obtained from the SNs were transmitted to the IoT LoRa gateway shown in Figure 11c. Subsequently, the buffered sensor node was waterproofed and submerged inside a pond. The readings for the temperatures and RSSIs at different distances were obtained until the submerged SN was out of range.

## 3. Results

In this section, the results obtained from the methods undertaken in the previous section shall be described.

### 3.1. Braid Antenna

#### 3.1.1. Simulations

**Figure 12:**
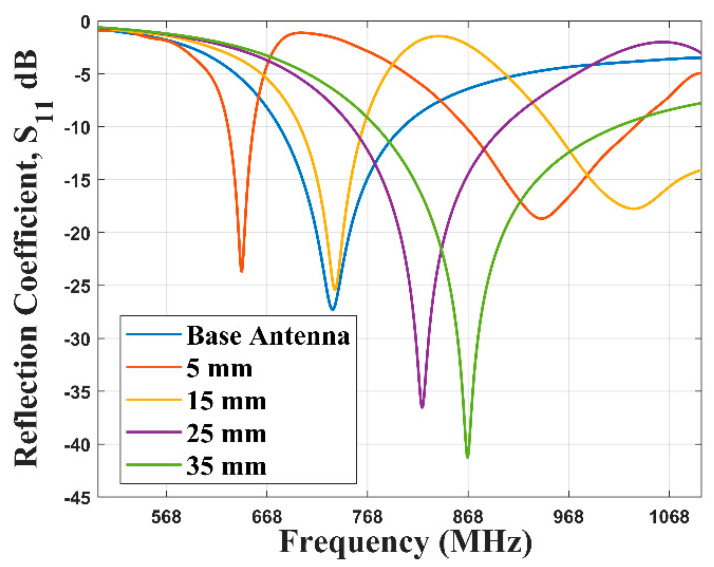
Reflection coefficient of braided antennas (with varied length).

The base antenna, as it can be seen from Figure 12, is resonant at 733 MHz. The 122 MHz bandwidth spans from 680 MHz to 802 MHz. The results from introducing the braid slots, scalene triangle element to the CPW, the two rectangular ground elements, and varying the rectangular length from 5 mm to 35 mm can be seen in Figure 12. This resulted in an upward translation of the resonant frequency which can be seen in Figure 12. The bandwidth for the 35 mm connector is resonant at 867 MHz and spans from 777 MHz to 1016 MHz, resulting in a 239 MHz bandwidth.

**Figure 13:**
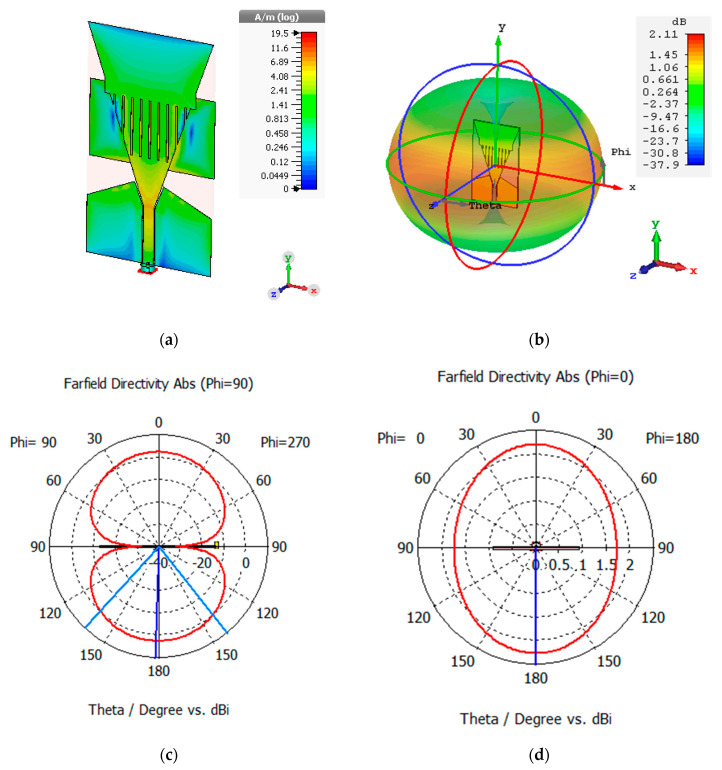
Optimal 35 mm antenna (**a**) surface current (**b**) 3D farfield radiation pattern (**c**) E-Plane radiation pattern (**d**) H-Plane radiation.

Some of the 35 mm connector braid antenna characteristics can be seen in Figure 13. The surface current can be seen from Figure 13a to be distributed along the slots introduced in the antenna in the form of braids and along the feedline. The antenna has an omnidirectional far-field radiation pattern, as can be seen in Figure 13b, which would enable the antenna to communicate with other sensor nodes in all directions. The antenna has a gain of 2.11 dBi. The antenna also exhibits omnidirectional radiation properties as could be seen from Figure 13d and a bidirectional radiation pattern in the E plane (Figure 13c).

#### 3.1.2. Fabrication

**Figure 14:**
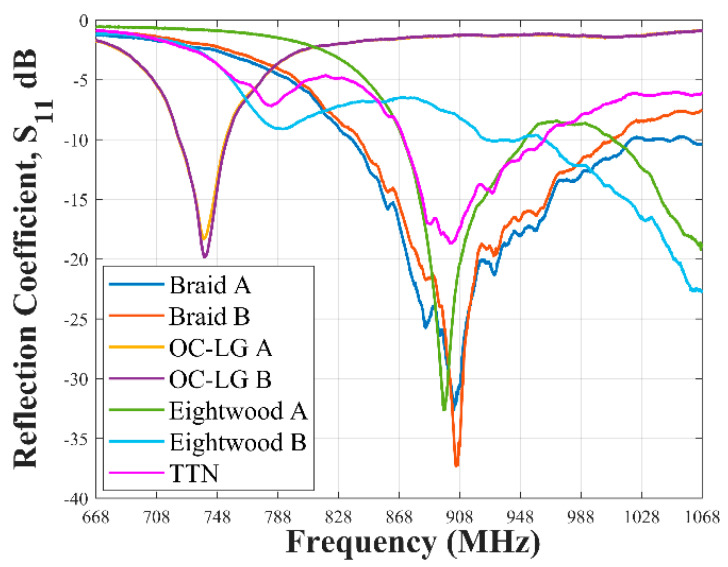
Measured S11 of braided and benchmarking antennas.

The 35 mm connector braided antenna was fabricated and its reflection coefficient, S11, was measured using an HP8753 VNA. The results can be seen in Figure 14. The TTN, Eightwood, and OC-LG antennas were all measured using the VNA. The −10 dB bandwidth for the OC-LG antennas were resonant at 740 MHz which spans from 723 MHz to 754 MHz resulting in a bandwidth of 31 MHz. Eightwood A Antenna has a bandwidth span of 81 MHz starting from 871 MHz to 952 MHz. Eightwood B starts from 925 MHz. The Things Network (TTN) antenna has a bandwidth that begins at 871 MHz and ends at 964 MHz, spanning a bandwidth of 93 MHz.

The fabricated braid A antenna has a bandwidth of 183 MHz, that starts from 838 MHz and ends at 1021 MHz. It has a centre frequency of 930 MHz. The braid B antenna had similar performance to the braid A antenna. It has a −10 dB bandwidth of 168 MHz that starts from 842 MHz and ends at 1010 MHz. As it can be seen from Figure 12, the simulated braid antenna’s −10 dB bandwidth spans from 778 MHz to 1016 MHz. This compares very well with both fabricated braid antennas.

#### 3.1.3. RSSI Tests

**Figure 15:**
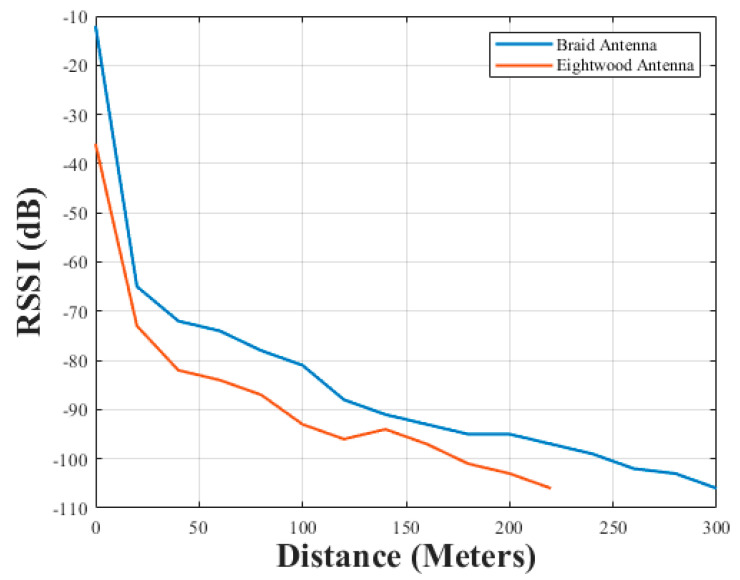
RSSI for antenna with respect to distance.

The result for the series of RSSI tests performed using both the braided antenna and Eightwood antenna can be seen in Figure 15 over 300 m.

The Eightwood antennas were selected because they exhibited better −10 dB bandwidth performance compared to the OC-LG antennas. The braid antenna could be seen to have better RSSI values over all the measured distance.

### 3.2. Oil-Impregnated Barrier

#### 3.2.1. Simulation

**Figure 16:**
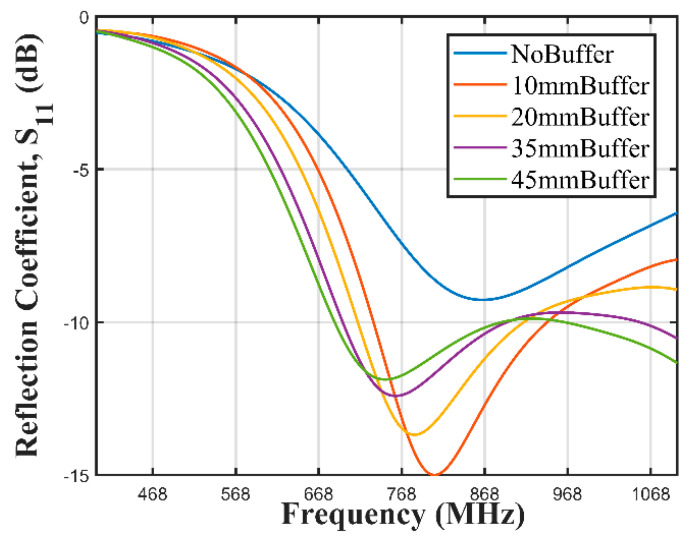
Braided antenna inserted in an oil-impregnated paper buffer.

Figure 16 shows the reflection coefficient of the antenna enclosed in the PLA container, placed on the buoy, which was in turn deployed in water. The Figure also shows the results obtained by introducing a barrier with oil-impregnated paper around the antenna and varying the buffer thickness from 10 mm to 45 mm.

#### 3.2.2. VNA Experiment

**Figure 17:**
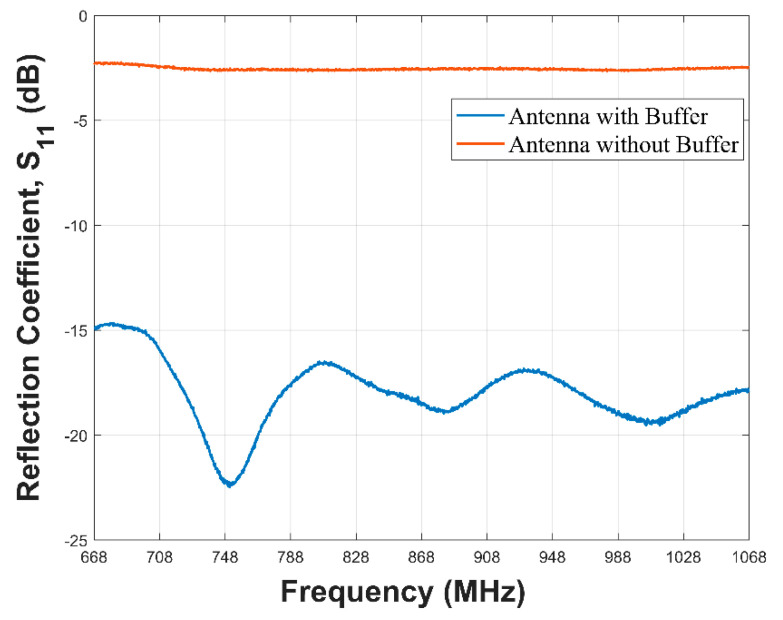
Measured results of buffered and barrierless antennas.

The VNA tests performed on both the buffered antenna and the barrierless antenna can be seen in Figure 17.

This could be seen to conform with the result obtained from the simulation with the barrierless antenna having no effective −10 dB bandwidth. It does not have a −10 dB bandwidth over the 868 MHz LoRa range of frequencies. The antenna with the buffer has better performance with a −10 dB bandwidth spanning the bandwidth of interest. This result is in agreement with the simulations results displayed in Figure 16.

#### 3.2.3. SN RSSI Tests

**Figure 18:**
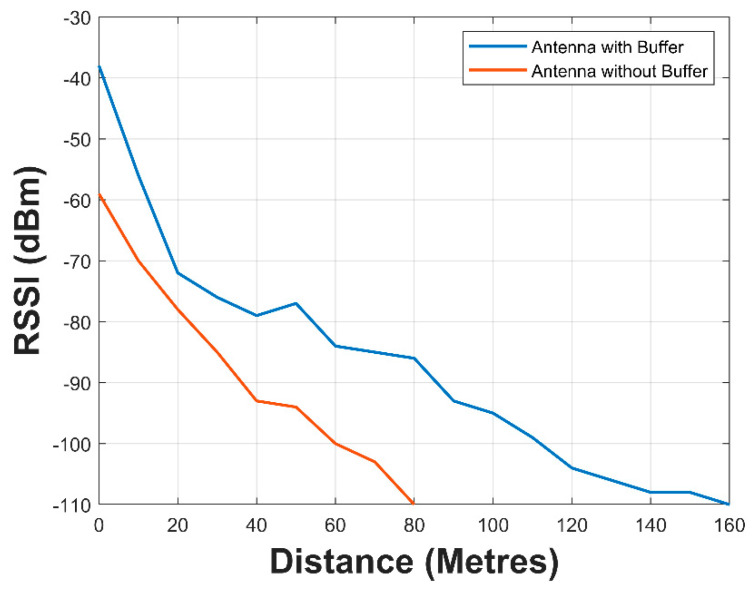
RSSI tests for buffered and barrierless antennas.

The result for the comparison of the SN antenna with and without the barrier could be seen in Figure 18. The RSSI of the water surface communication for the SN without the buffer could be seen to be worse than the one with the buffer. At 80 m, no signal was received from the SN without the buffer.

**Figure 19:**
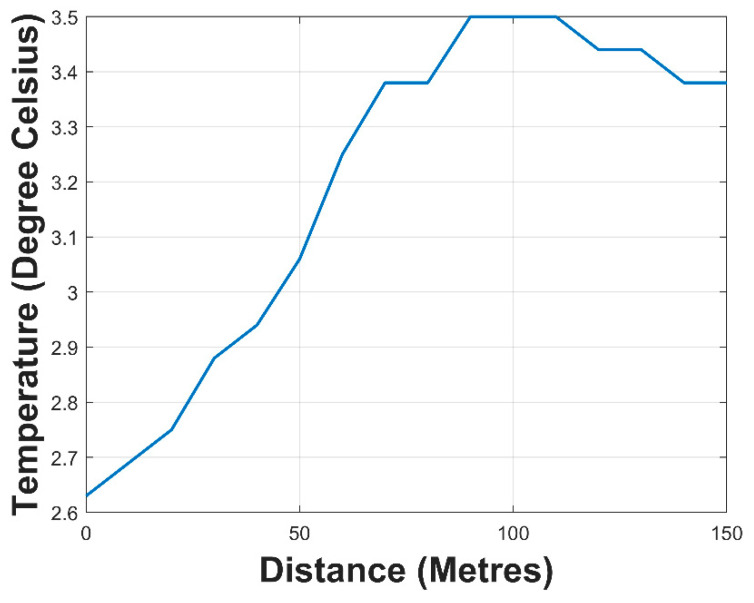
Temperature sensor data.

The one with the barrier had an extended reception up to 160 m. The temperature obtained during the submerged SN with buffered antenna test can be seen in Figure 19.

**Figure 20:**
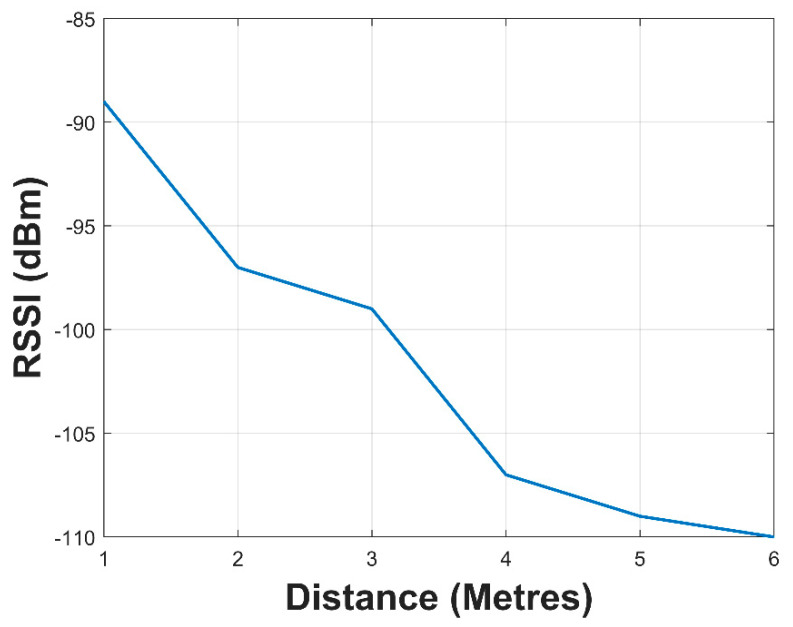
RSSI of submerged SN with buffered antenna.

When the SN with the buffered antenna was completely immersed in water, the values of the RSSI were obtained. The outcome of this experiment can be seen in Figure 20. The maximum distance attained was six (6) meters with an RSSI of −110 dBm. The range of operation of this wireless sensor network could be increased by placing sensor nodes at this distance to relay the sensor data in a multi-hop fashion. The SN at the water surface would then transmit the data to the data relay node for subsequent publishing on the internet.

## 4. Discussion

In this work, a design and implementation of a novel sensor node and data relay nodes were undertaken. Emphasis was placed on the design, fabrication, testing, and implementation of a novel braid antenna capable of both submerged and water surface communication at 868 MHz LoRa range of frequency. An oil-impregnated paper buffer region was incorporated around the braid antenna. We have shown that the introduction of this barrier substantially improved the performance of the degraded antenna when placed on and in water. We have been able to achieve substantial bandwidth improvement.

Initially, the base antenna was designed and simulated. However, its centre frequency was 733 MHz and had a frequency span from 608 MHz to 802 MHz. This does not fall within Europe’s ISM band of 868 MHz which the LoRa in the United Kingdom uses. By introducing braid slots to the patch antenna and a scalene triangle on the CPW, the performance of the antenna was improved. We could see from Figure 13 that increasing the rectangular element introduced to the patch antenna shifted the resonant frequency closer to 868 MHz. The connector length of 35 mm yielded the optimal antenna with respect to the S11 parameter.

The antenna had an omnidirectional radiation pattern which allows the SN to communicate with any other sensor node or gateway in every direction at the expense of directionality which would have yielded higher gain. The novel braid antenna was fabricated and the result as shown in Figure 15 shows the braid antenna A and B had the best bandwidth around the frequency of interest, 868 MHz, compared to the other antennas that were used for benchmarking. The result from the fabricated antenna were in agreement with the simulation results of the braid antenna. Both fabricated and simulated braid antennas spanned the frequency range of 842 MHz to 1010 MHz which falls within the LoRa ISM band in the United Kingdom.

The braid antenna pair and the pair of the best performing antenna among the off-the-shelf antenna underwent a received signal strength indicator test. The result obtained showed that the braid antenna exhibited better RSSI performance with respect to distance.

The reflection coefficient of the antenna deteriorated when it was enclosed in a PLA enclosure, fixed to a buoy, and placed on a water surface in the CST environment. The degradation worsened when the SN was submerged in water. This was obviously as a result of mismatch resulting from introduction of the water surface. The braid antenna was then sandwiched between two bunches of oil-impregnated paper. The buffer thickness was varied, and the buffer thickness of 10 mm showed the best reflection coefficient. This was chosen and the buffer was designed and implemented.

The reflection coefficients of both SN with the buffered and barrierless antennas were measured with a VNA and the result showed a superior performance exhibited by the SN with the buffered antenna. It had a −10 dB bandwidth spanning 668 MHz to 1068 MHZ while the SN with the barrierless antenna did not have an effective −10 dB bandwidth across the same frequency range. Received signal strength indicator tests were performed on both variants of the SN. The SN with buffered antenna sustained a communication over a distance of 160 m from the gateway node while the barrierless antenna was only capable of a communication at 80 m.

The SN with the buffered antenna was able to attain a communication range of 6 m with an RSSI of −110 dBm when submerged in water.

## 5. Conclusions

In this work, we were able to undertake a novel design, fabrication, measurement, and implementation of an antenna with dual ability of underwater and water surface LoRa communication at 868 MHz. The antenna was incorporated into a sensor node that was capable of transmitting temperature sensor data using RF communication underwater and over water surface communication to a data gateway node.

The integral part of the sensor node is the antenna which connects the node with the outside world. We designed, fabricated, and measured two braid antennas. Both antennas spanned between 842 MHz to 1010 MHz which falls within the 868 MHz LoRa Europe ISM band. The antennas conformed with the simulated antenna. Placing the antenna on water and underwater resulted in the degradation of the bandwidth resulting from mismatch from the water medium. We introduced an oil-impregnated paper barrier which resulted in ameliorating the mismatch. This was validated by measurements performed in the laboratory using a VNA where the buffered antenna was able to have an effective bandwidth from 668 MHz to 1068 MHz while the barrierless antenna did not have an effective −10 dB bandwidth across the same frequency range.

The practical systems were then deployed in an underwater environment and kept afloat over water surface to investigate the performances of the two systems. The SN with the buffered antenna was able to achieve a distance of 6 m underwater and 160 m over water surface communication to a data gateway node. The barrierless SN was only able to achieve 80 m over water surface communication.

With this information and these results, a wireless sensor network could be built using the sensor nodes developed in this research to cover larger areas and ranges by transmitting the data from the underwater sensor in a multi-hop fashion to the buoy sensor node at the water surface and subsequently relay the message to the IoT gateway for onward publishing on the Internet.

## Figures and Tables

**Table 1:** Dimensions of base antenna parameters.

Parameter	Symbol	Value
Substrate length	$S_{L}$	120 mm
Substrate width	$S_{W}$	70 mm
Substrate thickness	$S_{H}$	2.4 mm
Feed length	$F_{L}$	30 mm
Feed width	$F_{W}$	6 mm
CPW length	$CP_{L}$	15 mm
CPW width	$CP_{W}$	31.41 mm
Triangular side length	$T_{R}$	95.52 mm
Dielectric constant FR-4	$F\varepsilon_{r}$	4.3
Dielectric constant oil paper	$O\varepsilon_{r}$	3.87
